# Advances in Nano Neuroscience: From Nanomaterials to Nanotools

**DOI:** 10.3389/fnins.2018.00953

**Published:** 2019-01-15

**Authors:** Niccolò Paolo Pampaloni, Michele Giugliano, Denis Scaini, Laura Ballerini, Rossana Rauti

**Affiliations:** ^1^Neuroscience Area, International School for Advanced Studies (SISSA), Trieste, Italy; ^2^Department of Biomedical Sciences and Institute Born-Bunge, Molecular, Cellular, and Network Excitability, Universiteit Antwerpen, Antwerpen, Belgium; ^3^ELETTRA Synchrotron Light Source, Nanoinnovation Lab, Trieste, Italy

**Keywords:** neuroengineering, nanomaterials, nanoscience, neuroscience, nanotools

## Abstract

During the last decades, neuroscientists have increasingly exploited a variety of artificial, *de-novo* synthesized materials with controlled nano-sized features. For instance, a renewed interest in the development of prostheses or neural interfaces was driven by the availability of novel nanomaterials that enabled the fabrication of implantable bioelectronics interfaces with reduced side effects and increased integration with the target biological tissue. The peculiar physical-chemical properties of nanomaterials have also contributed to the engineering of novel imaging devices toward sophisticated experimental settings, to smart fabricated scaffolds and microelectrodes, or other tools ultimately aimed at a better understanding of neural tissue functions. In this review, we focus on nanomaterials and specifically on carbon-based nanomaterials, such as carbon nanotubes (CNTs) and graphene. While these materials raise potential safety concerns, they represent a tremendous technological opportunity for the restoration of neuronal functions. We then describe nanotools such as nanowires and nano-modified MEA for high-performance electrophysiological recording and stimulation of neuronal electrical activity. We finally focus on the fabrication of three-dimensional synthetic nanostructures, used as substrates to interface biological cells and tissues *in vitro* and *in vivo*.

## Introduction

NeuroNanoTechnology, an emerging treatment approach in neuroscience, is the manipulation of matter on a near-atomic size scale to produce new structures with atomic, cellular, or molecular functions (Huang et al., 2017) to manipulate or to heal damaged neural circuits. Nanotechnology is the science that deals with materials at nanoscale levels, and the collaboration of this field with neuroscience can transform basic science into novel materials and devices for the treatment and monitoring of the pathological condition in neurological disease. With their tiny dimensions, nanomaterials possess unique physiochemical properties such as conductivity, strength, durability, and chemical reactivity, and are already being used in electronics, sunscreens, cosmetics, and medicines (Yoshikawa and Tsutsumi, 2010; Huang et al., 2017). The advent of nanomaterials has also provided extraordinary opportunities for biomedical applications. Furthermore, nanomaterials are inert, which make them stable and allow binding to specific ligands thereby enhancing their use for targeted therapy (Mouhieddine et al., 2015; Huang et al., 2017). Applications of nanotechnology in basic and clinical neuroscience are only in the early stages of development, partly because of the complexities associated with interacting with neural cells and the mammalian nervous system. Despite this, an impressive body of research is emerging that hints at the potential contributions these technologies could make to neuroscience research (Silva, 2006). This review summarizes the diversity of nanomaterials and nanotools currently in use, underlying their recent applications in neuroscience. Specifically, it gives an overview of the current technologies, advanced imaging techniques and materials that are designed to better interact with neural cells, and describing the tremendous impact this nanotechnology might have on neuroscience research.

## Nanomaterials

Nanomaterials are defined as low-dimensional materials with building units smaller than ca. 100 nm at least in one dimension (Biswas and Wu, 2005; Farcau and Astilean, 2014; Dendisová et al., 2018). Nanomaterials are usually characterized by unique optical, electronic or mechanical properties, and are nowadays increasingly used in a multitude of applications such as medical (Ghosh et al., 2008; Chen et al., 2013; Oyefusi et al., 2014; Simões et al., 2015), cosmetic (Borowska and Brzóska, 2015; Patil et al., 2015), electronics (Kang et al., 2015; Zhou and Guo, 2015), machine engineering, and construction industry (Hincapié et al., 2015) or in the environmental field (Carpenter et al., 2015; Sharma et al., 2015). Due to their small size and advancements in synthesis methods, nanomaterials have many advantageous traits, such as high surface area-to-volume ratio, multi-functionality, site-specific delivery or targeting, controlled release, and versatility in enabling surface modification (Garbayo et al., 2014; Kumar et al., 2017). Therefore, nanomaterials can be used as vectors for drug delivery, as strategies for neuroprotection, as scaffolds for neuroregeneration, as modalities for neuroimaging and as devices for neurosurgery (Gilmore et al., 2008; Kumar et al., 2017). Engineered nanomaterials have a profound impact on a variety of applications, across diverse fields of research. In medicine, for instance, nanomaterials are interesting for diagnostics and implantable devices (Sahoo et al., 2007; Menon et al., 2013; Kumar et al., 2017) such as stents and catheters, which represent a large and critical market in the healthcare industry (Harris and Graffagnini, 2007). Nanoliposomes are some of the earliest nanomaterials engineered for drug delivery (Maurer et al., 2001; Kumar et al., 2017). These vesicles, composed of an aqueous core and one or several lipid or phospholipid bilayers, can be functionalized with monoclonal antibodies, which act as targeting ligands to enable receptor targeting to receptors expressed on tumor cells (Kumar et al., 2017). Liposome constructs functionalized with peptides specific to nicotine acetylcholine receptors on the BBB have been successfully used to deliver drugs such as doxorubicin, a chemotherapy drug, to glioma cells in an animal model (Shi et al., 2001). The ability to functionalize nanomaterials to achieve targeted therapy is perhaps one of the greatest advantages of nanotechnology, as it can potentially eliminate systemic toxicity, a conundrum in current chemotherapy (Kumar et al., 2017).

Novel medical devices with nanotechnological components aim at convenient real-time diagnosis of diseases. In addition, implantable devices with nanoscale features might cause lower irritation than conventional ones, while displaying improved functionality (Harris and Graffagnini, 2007). In Neuroscience, the increasing ease to design and synthesize nanomaterials has been exploited in basic and applied research (Giugliano et al., 2008; Pantic et al., 2015; Veloz-Castillo et al., 2016), pushing forward their potential for the field (Berger, 2016). For instance, the use of nanomaterials greatly improved the sensitivity and stability of microelectrodes used in electrophysiology (Keefer et al., 2008), of optical interfaces (Pisanello et al., 2016) and, more in general, is contributing to tackle the challenge of monitoring neuronal ensembles.

The use of nanomaterials presents also previously untapped source of potential in developing novel and superior neural tissue engineering materials and therapeutic strategies for CNS repair. Chief among these is the rapid development of nanotube scaffolds; with their extraordinary conductivity properties, such structures offer to support and even enhance native electrochemical activity by boosting the regenerative potential of the implant site (Kumar et al., 2015). Physically, these materials also mimic the tubular structures of axons and dendrites. These ideas have been implemented by several groups who have turned to carbon nanotubes (CNTs) based on their combination of electrical conductivity, mechanical properties, and comparable nanoscale dimensions to organic neuritis (Fattahi et al., 2014; Kumar et al., 2015).

The use of nanomaterials in neurosurgery has also the potential to improve patient prognosis and quality of life. Some areas of interest in nanomaterials within the context of neurosurgery include nano-electromechanical systems (NEMSs), laser-associated vascular anastomoses, nanoscaffolds for neural regeneration, biocompatibility of surgical prostheses, and nanowires (Khawaja, 2011; Mattei and Rehman, 2015; Kumar et al., 2017).

### Carbon-Based Materials

A fundamental key for the successful development of the nanotechnologies emerges from the constant improvement of the materials used to fabricate tools, devices, and scaffolds to be used in the nanotechnology-related fields. In this framework, a particular attention must be given to carbon-based nanomaterials, made of pure carbon with a variety of atomic hybridization or geometrical structures (Dresselhaus, 2012). To date, the three naturally occurring allotropes of carbon (diamond, amorphous carbon, and graphite) have been joined by allotropes deriving from synthetic processes (such as Graphene, Carbon Nanotubes, Fullerenes, and Nanodiamonds). Among all the family of carbon nanomaterials, Carbon Nanotubes (CNTs), and Graphene (GR) are currently the most popular and have been extensively studied for their excellent mechanical strength, electrical and thermal conductivity and optical properties. Due to their particular importance and strong impact in Nanotechnology-based research, we will discuss them more in details.

**Figure 1:**
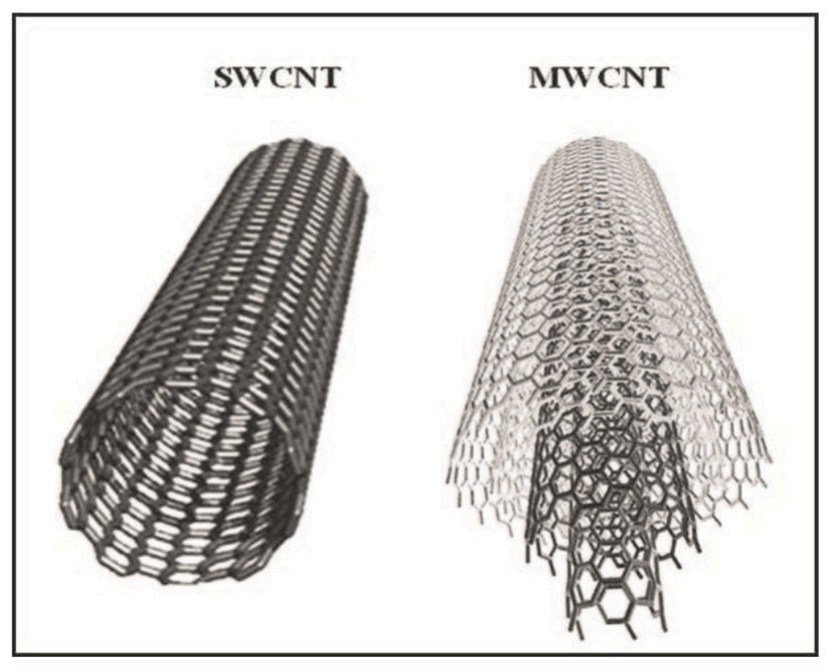
Schematic representation of a SWCNT, composed by just one graphene sheet, compared to a MWCNT, composed by more (three in this cartoon) graphene sheets (Choudhary and Gupta, 2011).

CNTs are allotropes of carbon discovered in 1991 (Iijima, 1991) and made up of one or more graphene sheets, rolled onto themselves to form small cylinders. Their diameter ranges from 1 to ~100 nm, and their lengths can reach up to ~15 microns. CNTs can be distinguished on the basis of their geometries. In the simplest case, CNTs can be formed by a single carbon-based sheet and are named Single-Walled (SWCNTs), while when two or more graphene sheets are involved, the CNTs are named Multi-Walled (MWCNTs; Figure 1; Choudhary and Gupta, 2011).

Chemical bonding of carbon atoms in CNTs are composed of sp^2^ bonds. This hybridization, not present in other allotropes of carbon, is essential for CNTs' peculiar mechanical strength and unique electrical conductivity (O'Connell, 2006).

Graphene is a single atomic layer of crystalline graphite, thus characterized by a bi-dimensional structure. It has a hybridized sp^2^ bonding, with three in-plane s bonds/atom and p orbitals perpendicular to the plane (Choi et al., 2010; Novoselov et al., 2012). It was only recently, in 2004, that bi-dimensional graphene was isolated and characterized (Novoselov et al., 2004). Such a discovery had a huge impact on various fields of science and technology, ranging from electronics to mechanics and to engineering, given the outstanding physical and chemical properties of this material, which has been named a “miracle” (Zumdahl and Zumdahl, 2014).

Carbon-based materials, especially CNTs and Graphene, have been widely used both in clinical and applied neuroscience research (Lovat et al., 2005; Mazzatenta et al., 2007; Cellot et al., 2009, 2011; Fabbro et al., 2012; Bosi et al., 2015; Rauti et al., 2016; Usmani et al., 2016). In fact, due to their peculiar physicochemical properties, CNTs and Graphene have been shown to best interact, and establish a peculiar cross-talk, with neuronal cells. To date the knowledge on the neuronal interactions with CNTs is much more exhaustive with respect to graphene, as the latter was introduced more than a decade later.

**Figure 2:**
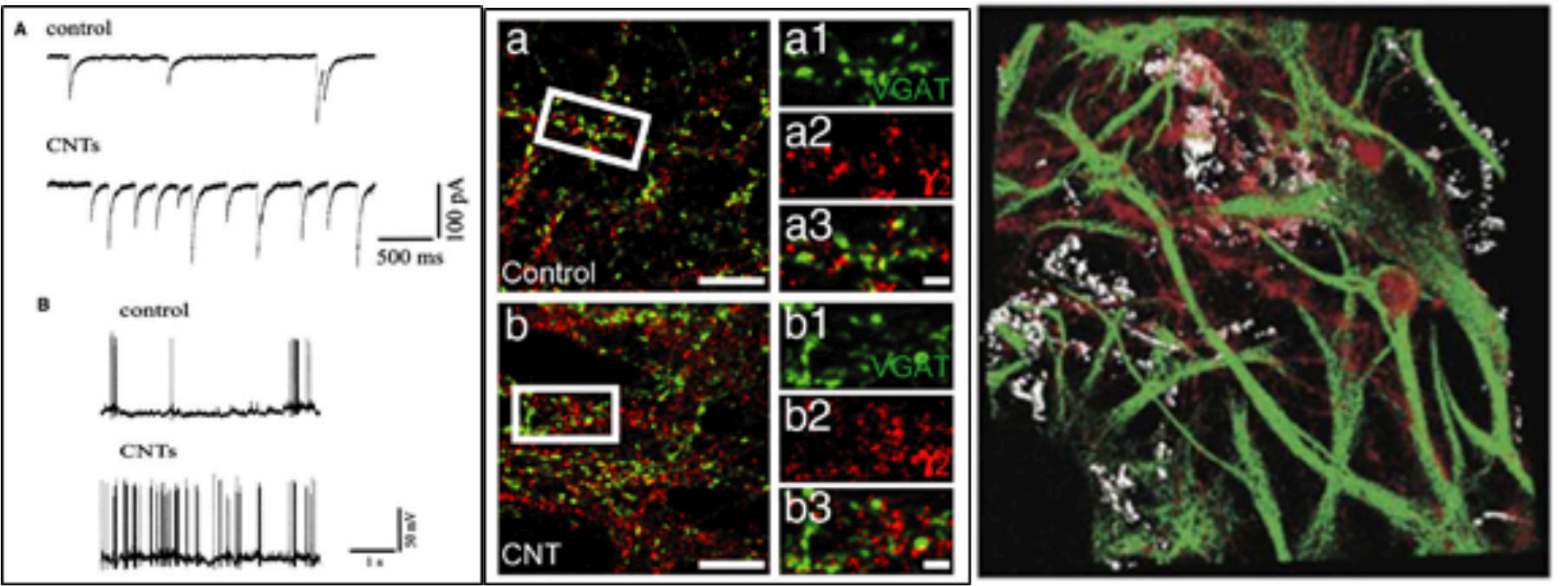
Neurons grown on a CNT substrates display **(Left)** increased spontaneous activity and firing (Reprinted with permission from Lovat et al., 2005, American Chemical Society) and **(Centre)** increased GABAergic synaptogenesis. On the right a confocal reconstruction of a 3D-MWCNT scaffold (in gray) with neurons (in red) grown suspended within a pore, and glial cells (in green) acting as a support (Bosi et al., 2015). **(A)** Spontaneous synaptic currents recorded from control and CNTs substrates. **(B)** Current clamp recordings from hippocampal neurons grown on control and CNTs substrates.

CNTs for instance, have been successfully used as substrates for neuronal growth (Mattson et al., 2000; Lovat et al., 2005). In those early studies it was shown that not only neuronal hippocampal cells were growing and surviving well on this material (Mattson et al., 2000), but also that neurotransmission was strongly potentiated compared to control conditions (Figure 2, left; Lovat et al., 2005), quantified in terms of an increased frequency either of spontaneous action potentials and of postsynaptic currents. Since then, the use of CNTs to interface cell growth has increased and has been further investigated. Today, we know for instance that CNTs affect neurons at single-cell level, likely establishing a neuron-substrate electrical coupling, and also increasing GABAergic and Glutamatergic synaptogenesis and heterogeneous short-term synaptic plasticity (Figure 2, middle) (Cellot et al., 2009, 2011; Pampaloni et al., 2017).

In organotypic spinal cord explants major cellular changes induced by CNTs were observed, such as an increased axonal outgrowth over two-dimensional CNTs substrates (Fabbro et al., 2012, Figure 3) or as an increased ability to establish a synchronized cross-talk across co-cultures (Usmani et al., 2016) over three-dimensional CNTs scaffolds. Cellot et al. (2017) showed also the ability of MWCNTs to interface cultured murine and human retinal neurons, pointing out the MWCNTs are promising materials also for the development of prosthetic devices aimed at restoring vision.

**Figure 3:**
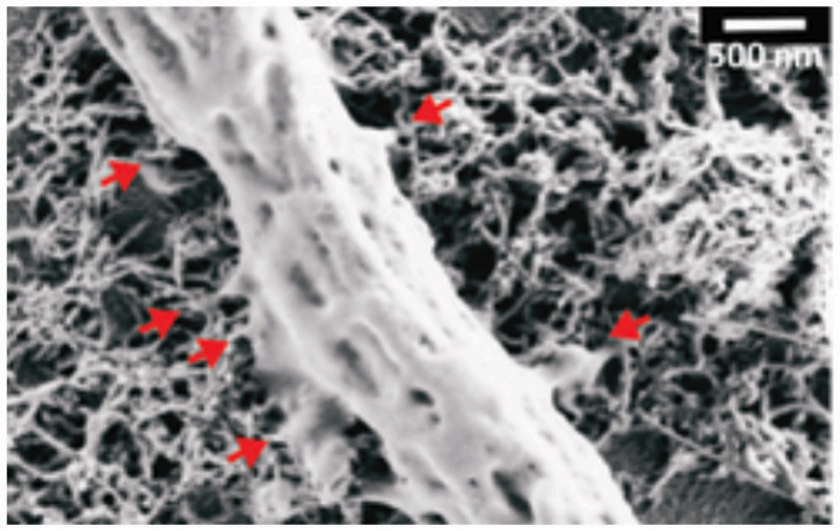
SEM micrograph showing a peripheral neuronal fiber establishing intimate contacts (red arrows) with the CNT carpet, suggesting that also in the case of spinal explants the ability of CNTs to couple tight to neural membranes (Modified with the permission from Fabbro et al., 2012, American Chemical Society).

Recently, the development of three-dimensional scaffolds further proved CNTs' ability to increase neuronal signals and boost synchronization *in vitro* (Bosi et al., 2015; Figure 2, right). Interestingly, CNTs three-dimensional scaffolds contribute to a more limited scar formation than control, when implanted in the rat primary visual cortex *in vivo* (Usmani et al., 2016). They implanted a pure MWCNT “sponge” (Usmani et al., 2016) or a “sponge” made by CNTs embedded into a polydimethylsiloxane (PDMS) matrix (Aurand et al., 2017). In both cases, the implant became well-integrated into the cortical tissue, with almost no scar formation surrounding the implant and a very modest gliosis reaction. Also, they showed that 4 weeks following the implantation, neural fibers penetrate inside the sponges thus indicating a very good biocompatibility of this material with the surrounding environment.

CNTs have been employed not only as substrates but also as detectors and devices: for instance CNT-based electronic transistor was fabricated as a field-effect transistor coated with SWCNTs and employed to detect the release of Chromogranin A (CgA) from cultured cortical neurons (Wang et al., 2007). Keefer et al. (2008) employed CNTs to improve the quality of electrophysiological recordings with conventional metal microelectrodes. Coating tungsten as well as stainless steel wire electrodes with CNTs, they showed that both signal recording and stimulation *in vivo* and *in vitro* could be improved by a decrease in the microelectrode electrochemical impedance and an increase in the electrical charge transfer. Also, CNT/gold composite Microelectrode Arrays (MEAs) were shown to boost the recordings of Field Potentials at all physiological signal frequencies (Keefer et al., 2008).

Graphene-based nanomaterials were also used as substrates for primary neuronal culture growth and were demonstrated to constitute a permissive interface on which neurons retain unaltered growth and signaling properties, key features for future carbon-based neuroprosthetics (Fabbro et al., 2016). Rastogi et al. (2017), showed that pristine graphene deposited onto a glass coverslip did alter neither the viability nor the general health of cultured primary neurons, assessed through the Tetramethylrhodamine ethyl ester (TMRE) assay evaluating the mitochondrial activity. These results pave the wave to exploit the unique features of Graphene for biomedical applications.

More recently, graphene was reported to tune the extracellular ion distribution at the interface with hippocampal neurons, key regulator of neuronal excitability. The ability to trap ions by graphene is maximized when a single layer graphene is deposited on substrates electrically insulated. These biophysical changes caused a significant shift in neuronal firing phenotypes and affected network activity (Pampaloni et al., 2018). Several other studies demonstrated the ability of graphene substrates to promote neurites sprouting and outgrowth (Li et al., 2011), to enhance neuron electrical signaling (Tang et al., 2013), and to reduce the inflammatory response (Song et al., 2014). It was also reported recently the ability of small graphene oxide nanosheets (s-GO) to interfere specifically with neuronal synapses, without affecting cell viability. In particular, in cultured neuronal networks, upon chronic s-GO exposure, glutamatergic release sites were sized down (Rauti et al., 2016).

Graphene is also considered emerging as a next-generation neuronal tissue engineering scaffolds to enhance neuronal regeneration and functional recovery after brain injury, being an electroactive material. Electrospun microfiber scaffolds coated with self-assembled colloidal graphene were implanted into the striatum or into the subventricular zone of adult rats (Zhou et al., 2016), while microglia and astrocytes activation levels were suppressed by functionalizing it. In addition, self-assembled graphene implants prevented glial scarring in the brain 7 weeks following implantation. Song et al. observed (Song et al., 2014) that 3D graphene foams supported the growth of microglia and showed good biocompatibility. Additionally, the 3D graphene foams facilitated the growth of neural stem cells and PC-12 cells (originated from neural crest) and proved that they can be used for neural repairing and neurogenesis. Growing neural stem cells on these substrates allows not only a more physiological condition but also a substrate that can be electrically stimulated. Neuronal dissociated hippocampal cultures, grown on 3D-Graphene scaffolds were also able to recapitulate two basic properties of the complexity of the brain: firstly, the coexistence of local and global electrical activity, and secondly, the existence of neuronal assembly with a degree of correlated electrical activity varying in space and time (Ulloa Severino et al., 2016). In a different strategy Martìn et al. built hybrid hydrogels with polyacrylamide and graphene. This study demonstrates that graphene improves the biocompatibility of 3D scaffold (Martín et al., 2017).

In order to promote the application of CNTs and graphene materials for biological interfacing applications, cell toxicity remains the most prominent issue to be addressed. Firstly, researchers found differences between the use of immobilized platforms or the use of free, unbound CNTs or Graphene particles. In fact, when used as substrates for *in vitro* studies, both pristine CNT and graphene were shown to have no major toxic effects on cell lines, dissociated primary cultures, or organotypic slice cultures (Lovat et al., 2005; Fabbro et al., 2012; Lee et al., 2015). Different is the situation regarding unbound particles, as both MWCNTs and SWCNTs may have toxic effects in their soluble forms, when not properly functionalized. This was shown to cause asbestos-like pathologies such as granulomas, DNA damage, altered expression of inflammatory genes, oxidative stress, and atherosclerotic lesions (Li et al., 2007). Because of their size, MWCNTs have unrestricted access to most parts of the lung, can reach highly vascularized alveolar regions, interstitium, and the pleural space, and exhibit a high degree of pulmonary biopersistence (Rahman et al., 2017). In experimental animals, exposure to MWCNTs via inhalation, aspiration or intratracheal instillation (Poulsen et al., 2013, 2016; Købler et al., 2015; Rahman et al., 2017) causes pulmonary inflammation, bronchiolar, and alveolar hypertrophy, interstitial fibrosis, and granuloma formation (Porter et al., 2010; Poulsen et al., 2015). The reported toxicity is mainly due to the capacity of MWCNTs and nanoparticles in general to enter into cells and disperse in the cytoplasm as demonstrated by Simon-Deckers et al. in human pneumocytes (Simon-Deckers et al., 2008; Baldrighi et al., 2016). However, it is important to note that no data on human cancer following exposure to MWCNTs is available at present. A few studies have reported tumors in animal models exposed to Mitsui-7, a type of long straight MWCNTs at high doses (Rahman et al., 2017). Based on the results of animal studies, the International Agency for Research on Cancer (IARC) has classified Mitsui-7 as possibly carcinogenic to humans (Group 2B) (Grosse et al., 2014). However, the underlying mechanisms are largely unknown and systematic research in this direction is urgently needed. For other MWCNTs types, data is not available. However, it should be noted, that a great contribution to these adverse effects could be led back to Fe, Ni, Co, and Y nanoparticles deriving from the CNTs synthesis, that are still present in variable amounts in raw CNTs samples. The careful removal of metal contaminants as well as chemical functionalization in fact leads to a drastic reduction of their toxicity (Pulskamp et al., 2007; Movia and Giordani, 2012; Baldrighi et al., 2016). Pondman et al. (2015) reported new novel methods to overcome the activation of classical inflammatory pathway that will lead to reduce inflammation and toxicity of CNTs by coating CNTs with recombinant globular heads (Pondman et al., 2015). Coated CNTs lack the collagen region of human C1q that will help escaping phagocytosis (Johnston et al., 2010; Pondman et al., 2015). Silva et al. (2014) studied the two different methods of administration (instillation vs. inhalation) and their effect on immune system with consideration of CNTs (dose, time, and physicochemical characteristics; Silva et al., 2014; Kobayashi et al., 2017). The study showed that original MWCNTs cause more inflammation than purified or functionalized MWCNTs (Silva et al., 2014). Choosing the right form of MWCNTs is another strategy to reduce toxicity (Kobayashi et al., 2017).

Similarly, it was demonstrated that pristine graphene induces cytotoxicity on murine macrophage-like cells (i.e., RAW 264.7 cells), upon depletion of the mitochondrial membrane potential, thus increasing the generation of intracellular Reactive Oxygen Species (ROS), and by triggering apoptosis upon the activation of the mitochondrial pathway (Seabra et al., 2014). Nonetheless, there are evidences that appropriate chemical modifications of CNTs and graphene can drastically decrease their associated hazard, making them biocompatible and, to some extent, even biodegradable. For instance, when carboxylated CNTs are left in a medium containing hydrogen peroxide, in the presence of the Horseradish Peroxidase enzyme, they are almost completely degraded after 10 days. Moreover, CNTs with the same functionalization were degraded by macrophages, likely thanks to the Myeloperoxidase activity (Bianco et al., 2011). Also, mice studies based on the uptake of graphene nanosheets, coated with polyethylene glycol (PEG), and on the subsequent photothermal treatment of tumors did not show any adverse toxic effects (Seabra et al., 2014).

Carbon nanotubes and graphene are the most studied carbon nanomaterials for neural interfaces, however Carbon Nanofibers (CNFs) are also attracting attention for their possible biomedical application for their electrical, chemical, and physical properties (Posthuma-Trumpie et al., 2012). CNFs based materials have been developed as electroconductive scaffolds for neural tissues to facilitate communication through neural interfaces (McCaig et al., 2005), not only providing physical support for cell growth but also delivering the functional stimulus. Recently, Guo et al. (2017) developed a polymer-based neural probe with CNFs composites as recording electrodes via the thermal drawing process (Guo et al., 2017). They demonstrated that *in situ* CNFs alignment can be achieved during the thermal drawing, which contributes to a drastic improvement of electrical conductivity by two orders of magnitude compared to a conventional polymer electrode. Its stable functionality as a chronic implant has been demonstrated with the long-term reliable electrophysiological recording with single-spike resolution and the minimal tissue response over the extended period of implantation in wild-type mice (Guo et al., 2017).

## Nanotools for Neuroscience

Nanoscience began with the technological ability to “observe” matter at the atomic scale. Understanding the molecular mechanisms by which neurons process and integrate synaptic inputs, as well as how these mechanisms are modified by activity, is a central challenge in nanoscience. Of particular interest are neuronal mechanisms that may be responsible for regulating signal localization and controlling the spatiotemporal regulation of biological functions in the brain.

Given the struggles of some techniques to the study of functional brain activity, the recent and rapidly advancing field of Neuronanotechnology presents a unique opportunity to confront these challenges and provide a platform to develop novel therapeutic strategies for neural diseases.

In the next sections, we will summarize some of the novel nanotechnologies (imaging nanotools, nanoparticles, devices, scaffolds, nanowires, MEA) highlighting some converging applications between nanotechnology and neuroscience with specific focus on how these technologies could further our understanding of CNS function and the progression of CNS disorders.

### Imaging Nanotools

In its early days, during the 1940s and the 60s, nanotechnology mainly focused on new types of microscopy techniques, such as the Scanning Tunneling Microscope (STM), able to image objects at their atomic level (sub-nanometric), the Transmission Electron Microscope (TEM), and the Scanning Electron Microscope (SEM), able to resolve images at the nanoscale with high-resolution. Later, in 1987, Atomic Force Microscopy (AFM) was introduced, where a sharp tip, mounted at the end of a flexible cantilever, is moved over the sample's surface and under a variety of configurations. This surface scanning is able to resolve the nanotopography of surfaces and map the spatial distributions of physico-chemical forces (Variola, 2015).

**Figure 4:**
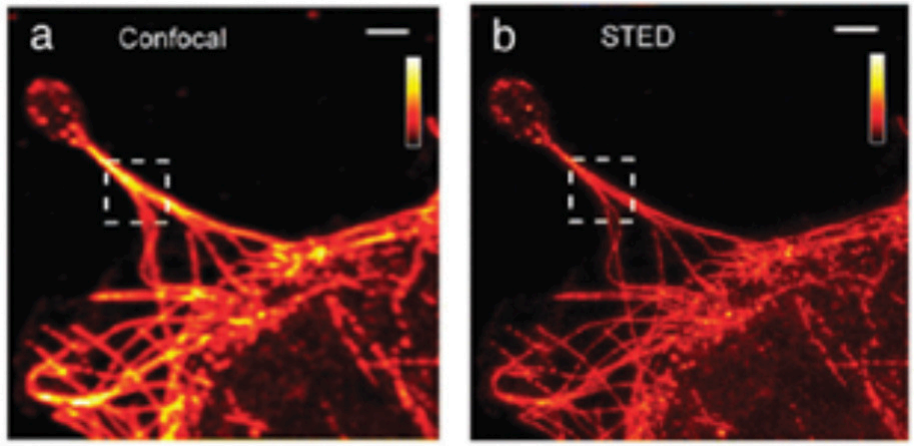
Samples of **(a)** classic confocal and **(b)** STED imaging of fibroblasts. Scale bar: 2 μm. (Modified with the permission of Variola, 2015, Published by the PCCP Owner Societies).

During the last decades, the field of microscopy further improved with the advances in Confocal Microscopy, in Total Internal reflection Fluorescence Microscopy (TIRFM), and others. During this time, these nanoscale imaging tools have shown tremendous potential for visualizing cellular biology, including details of cellular and even macromolecular structures, and they have been applied to measure fast nanoscale dynamics even on the single-molecule level. Recently, the Stimulated Emission Depletion (STED; Figure 4) microscopy has been introduced, where a system of paired synchronized laser pulses selectively inhibit the fluorescence in specific regions of the sample while sharpening the fluorescence at the focal spot, thus achieving resolution below the diffraction limit (Hell and Wichmann, 1994). STED microscope typically generates a maximum resolution of 20–50 nm, which has allowed the nanoscale topology of the cellular microcosmos to be imaged.

New optical imaging tools with nanoscale resolution, such as PALM (Pisanello et al., 2016) and STORM (Rust et al., 2006; Alivisatos et al., 2013) are helping scientists explore nanoscale objects within cells. These techniques can resolve structures in microscopic images with ~20 nm or better spatial precision. They thus promise to help uncover the organizational principles of macromolecular complexes within specialized cells of the nervous system (Alivisatos et al., 2013). As an example, recent work employing these techniques has revealed the dynamic behavior and organization of the actin cytoskeleton inside cells, which is relevant for understanding how neurons probe their involvement during neuronal outgrowth and in response to injury (Burnette et al., 2011; Alivisatos et al., 2013), and how they differentiate axonal processes (Alivisatos et al., 2013; Xu et al., 2013). These techniques also permit characterization of receptor clustering and stoichiometry at the plasma membrane under diverse conditions (Sengupta et al., 2011; Renz et al., 2012; Alivisatos et al., 2013) as well as protein organization inside synapses (Dani et al., 2010; Alivisatos et al., 2013), which are critical for understanding how synapses respond to changes in neuronal activity (Alivisatos et al., 2013).

### Nanoparticles

Other notable nanotechnology-based tools, routinely used in Neuroscience research and known for their outstanding features, are the Quantum Dots (Brus, 1983). These are semiconductor nanoparticles with unique optical and electronic properties (i.e., narrow emission spectra, resistance to photobleaching, high quantum yield) and ease of synthesis, and are used both for high-resolution imaging and as probes to label specific molecules or biological tissues (Jaiswal et al., 2004). Furthermore, their spectral properties make them ideal candidate to use as donors in Fluorescence Resonance Energy Transfer (FRET; Cooper and Nadeau, 2009), which is another important nano-technique based on the energy transfer from a donor chromophore to an acceptor chromophore, and mostly used to investigate molecular dynamics such as protein-protein interactions. Other significant nanotechnological advances include DNA nanotechnology, involving the synthesis of artificial nucleic acids for technological uses (Goodman et al., 2005), and a variety of artificial nanomaterials, such as fullerenes (Kroto et al., 1985), CNTs (Iijima, 1991), and graphene (Novoselov et al., 2004). By careful vapor deposition of carbon, with accurate control over geometry and bonding (Alivisatos et al., 2013), desirable electrical and mechanical properties of such nanomaterials can be controlled and modulated.

Among the promising nanotools, with applications in biomedical and basic research, colloidal gold Nanoparticles (AuNPs), need to be mentioned, whose wide range of diameters (i.e., 5–400 nm) alters their interaction with visible light and gives rise to a variety of different emission spectra, which encouraged their adoption for microscopy and bioimaging. Gold nanoparticles can also be coated with molecules and then used as therapeutic-agent delivery or as sensors in diagnostic applications.

Recently, the group of Francisco Bezanilla in Chicago exploited the AuNPs' ability to transduce light into heat as a neuronal stimulation technique. While employing 20 nm-sized AuNPs, they coupled them to a synthetic molecule (i.e., Ts1) able to bind sodium channels without blocking them. Once green laser pulses were delivered to biological samples with functionalized AuNPs, the light caused a transient and local increase in temperature that evoked a transient increase in the membrane capacitance. Such an increase caused in turn a membrane potential depolarization, leading to the firing of action potentials (Carvalho-de-Souza et al., 2015). This technique has impressive potential applications, as it would allow the light-driven neuronal stimulation with no need of viral transduction (as in Optogenetic), being minimal invasive when compared to traditional electrophysiology.

### Devices

Several generations of devices have been introduced over the last few years, such as the Metal Nanoelectrodes (Zhu et al., 2014), the functionalized quantum dots (Silva, 2006), or the Carbon-Nanofibers-based Micro- and Nanodevices (Zhang et al., 2012). Here we focus our discussion to nanowires, Microelectrode Arrays (MEAs) and scaffolds, given the role played by these devices in Neuroscience for applied and basic research applications. Furthermore, as these devices display micro- and nano-sized features, advances and progresses proceed hand in hand with the advances in the field of nanomaterials.

### Nanowires

Nano-needles and Nanowires (NWs), are artificial nano- or micro-sized “needles” that can provide high-fidelity electrophysiological recordings if used as microscopic electrodes for neuronal recordings. By such devices, recordings and stimulation of neuronal activity was shown both *in vitro* and *ex vivo*, in a highly scalable fashion (Robinson et al., 2012; Alivisatos et al., 2013, Figure 5).

**Figure 5:**
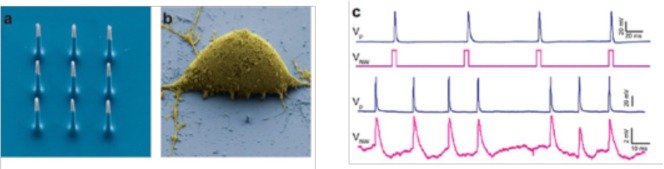
**(a)** SEM image of the nine silicon nanoneedles that constitutes the active region of a 3D-NEA. **(b)** SEM micrograph of a rat cortical neuron on top of an electrode pad; **(c)** example of stimulation and recording of rat cortical neurons showing that Action potentials (upper blue trace, measured by a patch pipette) could be reliably stimulated by voltage pulses applied to the nanoelectrodes (lower magenta trace; reprinted with permission from Alivisatos et al., 2013, American Chemical Society).

Nano-needles have been proposed for other techniques, such as AFM. For instance, Obataya et al. (2005) sharpened AFM tips into ultrathin needles of 200–300 nm in diameter and shown their ability to penetrate the cell nucleus, and offering a proof of principle of fine subcellular surgery in living cells.

Among diverse nanoscale architectures, NWs are highly functional structures and offer unique properties due to their dimensionalities and electronic properties (Cui and Lieber, 2001; Cui et al., 2001,b, 2003; Wu et al., 2004; Vidu et al., 2014). Especially, the electrical conductivity through NWs is greatly affected by the biological/chemical species adsorbed on their surface. Hence, NWs are effectively used to develop nanoscale devices with enhanced sensing performances (Ahmad et al., 2018). One of the most powerful and versatile platforms based on NWs devices has emerged to build functional interfaces for biological (including neurons) systems. NWs are non-invasive (highly local) probes of neuronal projections; individual NWs devices are becoming optimal for interfacing with neurons due to the fact that the contact length along the axon (or the dendrite projection crossing a NW) is just about 20 nm (Vidu et al., 2014).

A wide class of NWs have been developed, ranging from NWs based on classic semiconductors, such as silicon NWs (Chen et al., 2006; Goncher et al., 2006; Yajie et al., 2008; Vidu et al., 2014), GaP (Dujavova-Laurencikova et al., 2013), CdS, and ZnS (Barrelet et al., 2003; Vidu et al., 2014), oxide nanowires MgO (Yin et al., 2002), Cu_2_O (Jiang et al., 2002), SiO_2_ (Zheng et al., 2002), Al_2_O_3_ (Xiao et al., 2002). Recently, it has been shown that epitaxially grown gallium phosphide (GaP) NWs have beneficial properties for neuronal interfaces such as improved cell survival (Hällström et al., 2007; Suyatin et al., 2013) and improved cell adhesion (Prinz et al., 2008; Suyatin et al., 2013). GaP nanowires can be synthesized with a high aspect ratio (>50), very little tapering and exceptional control over their position and geometry, compared to other material nanowires (Suyatin et al., 2009, 2013).

Ferguson et al. developed nanowires grown over microwire electrodes for intracellular recording of action potentials within rat hippocampal slices. Their results indicated improved recording capabilities of intracellular neuronal activity that could allow for extensive recording of chronic activity from intact neural tissues and mammalian brains. They suggested that the development of a nanoelectrode platform with supporting microwire structures could be well-suited to *in vivo* studies of neural tissue (Ferguson et al., 2012; Ajetunmobi et al., 2014).

Nanowire field-effect transistors (NWFETs) comprising chemically synthesized semiconductor nanowires as functional channels, increasingly represent an effective method for subcellular recording between biosensors and biological systems (Cui et al., 2001,b; Zheng et al., 2004; Duan et al., 2013; Ajetunmobi et al., 2014; Vidu et al., 2014). The earliest use of NWFETs for extracellular recording of neural tissue involved the use of surface patterning of poly-L-lysine onto NWFET device sensors. NW-based FET device can be designed into a device array; neuron growth over dense NWs device arrays is usually achievable nowadays (Patolsky et al., 2007). Thus, interfacing ensembles of NWs inputs and outputs to different neural networks and neurons enables the implementation of stimulation, inhibition, or reversibly blocking signal propagation through specific pathways (Vidu et al., 2014). Besides single NW-based FET devices or arrays of NW-based FET devices used for investigating neuronal activity, the NWs are also used to design and build NWs- based electrodes for neural recordings in the brain.

Recently, silicon-based three-dimensional vertical nanowires electrode arrays (VNEA), consisting of 16 stimulation/recording sites, have been developed and shown to enable high fidelity recordings and stimulation of up to hundreds of individual rat cortical neurons (Robinson et al., 2012).

The first functional testing *in vivo* of a NWs-based device was performed during acute recordings in the rat cerebral cortex, where the NWs were used as a backbone for a metal nanostructured electrode with a three-dimensional (3D) structure. This electrode design opened the development of a new model system, with the prospect of enabling more reliable tissue anchoring as well as a more intimate contact between the electrode and the neurons (Xie et al., 2010; Vidu et al., 2014) furthering research on the functionality of nanostructure-based neuronal interfaces *in vivo*, given the better electrode-cell electrical coupling (Hai et al., 2010; Xie et al., 2012; Vidu et al., 2014).

Suyatin et al. (2013) were the first to achieve a functional testing of a GaP-NW-based electrode by performing acute recordings in the rat cerebral cortex (Suyatin et al., 2013). With this electrode design, they provide the first step of the development of a new model system for further research on the functionality of nanostrucuture-based neural interfaces *in vivo*, with the aim to provide a better electrode-cell electrical coupling (Robinson et al., 2012; Xie et al., 2012; Suyatin et al., 2013).

### Nano-Modified MEAs

**Figure 6:**
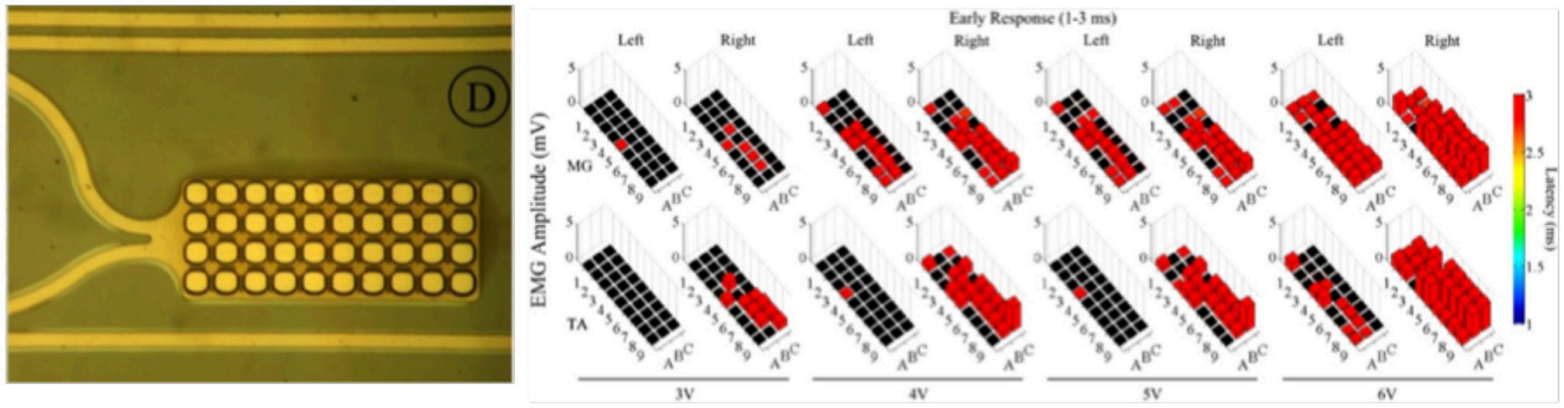
**Left**: Zoomed-in view of a single electrode along with the platinum traces; **Right**: Effects of low frequency monopolar stimulation on the ER. Early responses (1–3 ms latency) recorded in the MG (top row) and TA (bottom row) bilaterally during low frequency (1 Hz) monopolar stimulation (3–6 V) at each electrode on the array. The height of each bar indicates the amplitude and the color indicates the latency of the response. The black box indicates a case where no response was recorded for that particular window (Modified with the permission from Gad et al., 2013).

Micro- and nanotechnologies have been recently combined together, opening new routes for the use of substrate-integrated arrays of microelectrodes (MEAs) in Neuroscience. Critically, MEAs offer considerable flexibility in culture preparation, experimental design, and high-throughput approaches when compared to conventional electrophysiological techniques (Morin et al., 2005; Ajetunmobi et al., 2014). MEAs have been pioneered in the 70 s (Pine, 2006), where early microfabrication techniques were used to obtain devices to be electrochemically coupled to neurons *in vitro* and *in vivo*. The use of MEAs has been demonstrated for both recording of bioelectric signals as well as for electrical stimulation of neurons, and shown to successfully enable a non-invasively monitoring of the activity of cultured networks (Gawad et al., 2009; Hales et al., 2010; Obien et al., 2014) as well as target CNS regions *in vivo* (Gad et al., 2013; Spira and Hai, 2013; Figure 6). Ideally, all materials used in MEA fabrication should have high biocompatibility, excellent electrical properties for a high signal-to-noise ratio of signal detection, transparency for the direct cell observation, while being cost-effective (Liu et al., 2012). However, the most crucial and desirable feature is the close proximity and intimate mechanical contact between neurons and devices. This is known to lead to dramatic improvements in the electrical coupling between the device and the neurons, defined as the ratio between the maximal signals detected by the device and the maximal transmembrane potential of an excitable cell (Spira and Hai, 2013). The key advantage of MEA technology is the possibility to increase the spatial resolution of conventional electrophysiological techniques, enabling the recordings of simultaneous extracellular signals at spatially distinct sites in a high-density arrangement. One of the very latest technological achievement of microelectronic integration in the field is represented by the Neuropixel probe (Jun et al., 2017), where a very high density of ~1,000 active electrical contacts is packed in a single shank device, suitable for *in vivo* implants. However, more conventional devices, such as the commercial MED64 system, have been extensively used and presented in the literature. These have lower microelectrode counts, e.g., arranged in 8 × 8 layouts of 64 microelectrodes, where each passive metal contact is composed of platinum black, gold, and nickel, microfabricated on a glass substrate, and connected via indium tin-oxide (ITO) strip conductors to external contacts (Liu et al., 2012).

As an alternative to calcium- and voltage-sensitive dyes imaging, MEAs have been successfully used to investigate network dynamics on both dissociated cultures and acute brain explants. For instance, performing MEA recordings on hippocampal brain slices, Zhao et al. (2009) could correlate peripheral persistent nociception to changes in temporal and spatial plasticity of synaptic connections and of function within the hippocampal formation. In another study, Heidemann et al. (2015) cultured organotypic slice co-cultures of the rat spinal cords onto MEAs and characterized the spatial and temporal patterns of their spontaneous activity and the degree of synchronization between the two slices.

The recent trend in higher density electrode arrays on MEA devices has led to the implementation of novel nanotechnology-based approaches to enhance MEA performance for neuronal recording and stimulation and new features have been developed to enable precise cell growth and guidance on novel nano-enhanced arrays (Heim et al., 2012; Ajetunmobi et al., 2014). In particular, nanoscale grooves developed through photolithographic techniques have been successful in enhancing neuronal interaction with array substrates (Ajetunmobi et al., 2014). For example, in a study of axonal outgrowth on nano-imprinted patterns, Johansson et al. (2006) developed nanoscale grooves with depths of 300 nm and varying widths of 100–400 nm on polymethylmethacrylate (PMMA)-covered silicon chips. They monitored the growth of mouse sympathetic and sensory ganglia cultured in medium containing 25 ng/ml of nerve growth factor to stimulate axonal outgrowth. Using immunocytochemistry and scanning electron microscopy, they found that axons displayed contact guidance on the patterned surfaces but grew preferentially on ridge edges and elevations in the patterns rather than in grooves (Johansson et al., 2006; Ajetunmobi et al., 2014).

During the last few years, the discovery of new nanomaterials allowed the construction of nano-modified MEAs, in order to improve cell adhesion properties and cell-to-substrate MEA. David-Pur et al. (2014), for instance, employed conductive Carbon Nanotubes (CNTs) films, embedded in a polymeric support, to fabricate flexible MEAs aimed at neuronal recording, and stimulation. The CNT-electrodes displayed a very large capacitance and low electrical impedance, enabling highly efficient neuronal recording, and stimulation as demonstrated in chick retinas. Shein and co-workers presented novel electrode arrays composed of cell-appealing CNT islands of microelectrodes coated by a layer of dense and entangled CNTs (Shein et al., 2009; Ajetunmobi et al., 2014). They showed that the CNTs islands strongly attracted and anchored dissociated primary rat neurons to pre-defined locations and enabled the formation of stable sub-networks on electrically active recording sites (Ajetunmobi et al., 2014). They concluded that CNT-coated electrodes were well-suited to assist interfacing between electrically active biological cells and conventional electronic systems (Shein et al., 2009; Ajetunmobi et al., 2014). In another study, Wang et al. presented a MEA neural interface employing vertically aligned multi-walled CNT pillars as micro- electrodes. They demonstrated the effectiveness of their platform using hippocampal sliced cultures grown on the CNTs-modified MEA device and revealed superior charge injection limits than compared to standard platinum electrodes (Wang et al., 2006; Ajetunmobi et al., 2014). Similarly, Yu et al. tested vertically aligned carbon nanofiber electrode arrays for their potential to record electrophysiological activity and reported the stimulation and extracellular recording of spontaneous and evoked electrical activity in organotypic hippocampal slice cultures. Their results suggested the potential use of such a platform in improving electrophysiological studies of neuronal populations by enabling multimodal recordings at high spatial resolutions (Yu et al., 2007; Ajetunmobi et al., 2014).

MEAs have also been investigated as high quality, chronic *in vivo* neural interfaces and the nano-modification techniques have found increasing use for improving implantable MEA devices (Kipke et al., 2008). Keefer et al. reported the use of CNTs covalently attached to either amine-functionalized gold electrodes or electropolymerized with the conductive polymer polypyrrole to the electrode surface. Both strategies were used for *in vivo* study of the rat motor cortex and the monkey visual cortex and showed reduced impedance and noise, enabling simultaneous measurements of local field potentials and spike activity from the same electrode site (Keefer et al., 2008; Ajetunmobi et al., 2014). Similarly, Park et al. (2014) developed a transparent, carbon-layered microelectrode array (CLEAR) made of graphene. They implanted such a device in the rat cortex and, in addition to direct optogenetic stimulation and fluorescence imaging at the microelectrode sites, they were able to record neural signals with the same quality of the platinum-based MEAs, with comparable longitudinal tissue responses. Graphene was also used by Kireev et al. (2017), to build flexible MEAs. These authors showed how their Graphene microelectrodes (GMEAs), fabricated in a dense array on a flexible polyimide substrate, displayed excellent robustness, and low-noise recordings when combined to rat-derived acute heart tissue and cardiac muscle cells (Kireev et al., 2017).

### 3D Scaffolds

Another important recent application of nanotechnology is that of tissue-engineering scaffolds. These are three-dimensional synthetic nanostructures used as substrates to interface biological cells or tissues *in vitro* and *in vivo* (Cooper and Nadeau, 2009). Due to their characteristic 3D structure, the ease of surface functionalization, and to the variety of forms and materials, ranging from CNTs (Bosi et al., 2015) to biomaterials (Londono and Badylak, 2015) and hydrogels (Kunze et al., 2009; Nagai et al., 2012), scaffolds became highly successful in a vast range of fields, from regenerative medicine (Hosseinkhani et al., 2014; Carballo-Molina and Velasco, 2015; Londono and Badylak, 2015), to biomedical applications (Gupta et al., 2014) to neuroscience basic (Bosi et al., 2015; Carlson et al., 2016), and applied research (Cavallo, 2015). In tissue engineering, for instance, biomimetic scaffolds are emerging as a possible treatment after neural tissue degradation or injury (O'Brien, 2011; Tsintou et al., 2015), as hydrogel scaffolds were shown to promote axonal regeneration after a peripheral nerve lesion (Carballo-Molina and Velasco, 2015). In fact, hydrogel scaffolds could soon become a viable alternative to conventional drug-release systems. During the gelification process, it is possible to incorporate different types of molecules or biological cells into the gel structure, facilitated by the high quantity of water that enables the uptake and diffusion of soluble molecules (Nagai et al., 2006; Carballo-Molina and Velasco, 2015).

Recently, with the development of self-assembling peptide nanofiber scaffolds (SAPNS), a new protective, therapeutic strategy for intracerebral hemorrhage (ICH) has emerged (Kumar et al., 2017). One study evaluated ICH-related brain injury and functional recovery by observing the effects of hematoma aspiration and intrastriatal administration of RADA16-I. Intracerebral delivery of SAPNS into the hemorrhagic lesion of a rat model of ICH replaced the hematoma and reduced acute brain injury. With SAPNS functioning as a biocompatible material in hemorrhagic brain cavities, the formation of brain cavities was reduced, and an improvement in recovery of sensorimotor function was also observed. The local delivery of SAPNS as a treatment for ICH-related brain injury may allow better repair of ICH brain damage and improved recovery rates (Sang et al., 2015; Kumar et al., 2017).

**Figure 7:**
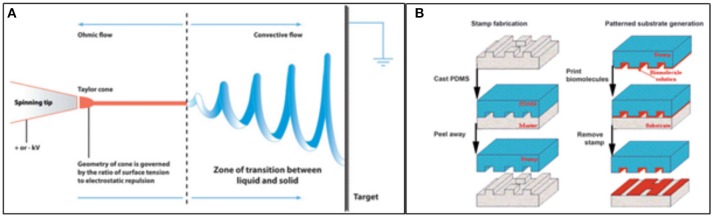
Techniques employed in patterned scaffold generation. **(A)** Diagram showing the basis of the Electrospinning technique; **(B)** Schematics of the steps of microcontact printing, showing the creation of a PDMS mask and the deposition of biomolecules onto a substrate using the stamp (Cooper and Nadeau, 2009).

When the patterning of a substrate is required, electrospinning and microcontact printing (Figure 7) are the most widely employed technique. Electrospinning is a fabrication method that uses electric charges to form fine fibers from polymers solution, as demonstrated with both synthetic [i.e., polycaprolactone, poly(glycolic acid)] and natural polymers, such as collagen. During the process, a polymer solution is passed through a tip and subjected to high voltage that charges the conductive liquid. Liquid droplets are then stretched beyond the expected shape by electrostatic repulsion, into a resulting surface known as the Taylor's cone. At a critical point in space, the liquid erupts in a stream. As the jet dries, charge migrates to the surface of the fiber and the mode of current flow changes from conductive to convective. The jet is then elongated by whipping, caused by electrostatic repulsion, and captured on an electrically grounded collector surface. Deposition of the stream onto the edge of a rotating disk produces aligned nanofibers (Figure 7A; Cooper and Nadeau, 2009).

Microcontact printing, on the other hand, is highly accessible and produces substrates with controlled patterning. During a first step, a “hard stamp” based on a Si wafer is fabricated by means of a printed photolithographic mask containing the pattern of interest. Then, a soft stamp is made from poly-dimethylsiloxane (PDMS) from the mask. The PDMS may be then enriched with proteins, growth factors, and other molecules (Figure 7B; Cooper and Nadeau, 2009).

As mentioned above, electrospinning is mostly used to produce Nanofibers with a diameter lower than 1,000 nm and mainly employed in wound healing and tissue repair (Cetin et al., 2012). Nanofibers have been employed in a variety of scaffolds and interfaced with many different biological samples, as electrospun meshes generally comprise of non-woven fibers with diameters in the range of hundreds of nanometers, and highly interconnected pores that are tens of micrometers in diameter. The large surface area–volume ratio of such fibrous meshes also ensures abundant area for cell attachment, allowing a higher density of cells to be cultured than with flat, two-dimensional, surfaces. The morphological resemblance of electrospun nanofibers to native ECM suggests their natural application as a supportive matrix for creating scaffold constructs for stem cells (Lim and Mao, 2009). Electrospun poly(ε-caprolactone) (PCL) scaffolds have been shown to be biocompatible, as they well-integrated in the caudate putamen of the adult rat brain, with no evidence of microglial encapsulation after 60 days *in vivo*, with visible neuronal processes penetrating into the scaffold as another evidence of a successful neuronal-scaffold integration (Nisbet et al., 2009).

In order to obtain sustained release of drugs *in situ*, scaffolds in the form of nanofibers were found to hold potential as implants for neurological therapies, such as Parkinson's disease. Initial attempts for site-specific delivery of dopamine to minimize its peripheral side effects were focused on designing and biometric simulation of a prototype nano-enabled scaffold device (NESD) comprising of an alginate scaffold embedded with dopamine-loaded cellulose acetate phthalate (CAP) nanoparticles (Pillay et al., 2009; Das et al., 2016). The device was implanted in the parenchyma of the frontal lobe of rats and was found to deliver dopamine over 30 days with 10-fold more dopamine in CNS as compared to systemic concentration.

Hydrogel scaffolds, beyond tissue regeneration, have been also demonstrated as optimal substrates for neuronal growth. Hanson Shepherd et al. (2011) developed scaffolds of poly(2-hydroxyethyl methacrylate) (pHEMA) with varying architectures on which primary hippocampal neurons were grown. Authors then showed that neuronal organization was strongly reflecting the scaffold spatial organization, proving that neurons were able to finely sense mechanical spatial cues and rearrange their network organization accordingly. Kunze et al. instead developed a microfluidic PDMS-based device, fabricated with different agarose/alginate parallel layers and thus resembling the cortex neuronal layers. In such layers, dissociated cortical neurons could properly grow and extend their neurites across neighboring layers. As the agarose/alginate hydrogel could potentially be populated by distinct cell types and by drug compounds, this scaffold represents a useful tool to address questions on neural networks development and for drug testing experiments (Kunze et al., 2009).

In another work, it was shown that laminin-functionalized nanofibers in 3D hyaluronic acid hydrogels enabled a significant alignment of neuronal neurites along the nanofibers, while significantly increasing the distance over which neurites could extend (McMurtrey, 2014). Another example of the potential of these substrates as an optimal tool for investigating neuronal ensembles, has been given by Huang et al. (2013), who demonstrated that three-dimensional collagen-based scaffolds promoted the differentiation of neural stem cell (NSC) into mature neurons earlier than what achieved in neurospheres cultured in suspension. The same kind of cells were shown to grow not only on hydrogel-made scaffolds, but also on porous scaffolds of graphene: in this case, it was seen that not only the scaffold supported the NSC growth, keeping the cells at an active proliferation, but also that cells established a good electrical contact with the graphene foam, as the authors were able to electrically stimulate the cells via the scaffold itself (Li et al., 2013).

The combination of NSCs with nanofiber scaffolds revealed also great potential in regenerating axons through the formation of a growth-supportive microenvironment, and is considered to hold great therapeutic potential for the treatment of CNS injury and disease (Martino and Pluchino, 2006; Das et al., 2016). Poly(lactic-co-glycolic acid) (PLGA) scaffolds containing pores for axonal guidance, and an underlying layer seeded with NSCs, were implanted in rats with spinal cord hemisection lesions (Teng et al., 2002). Implantation of this scaffold led to axonal regeneration and a functional recovery which was superior to that seen in rats implanted with the scaffold or NSCs alone.

**Figure 8:**
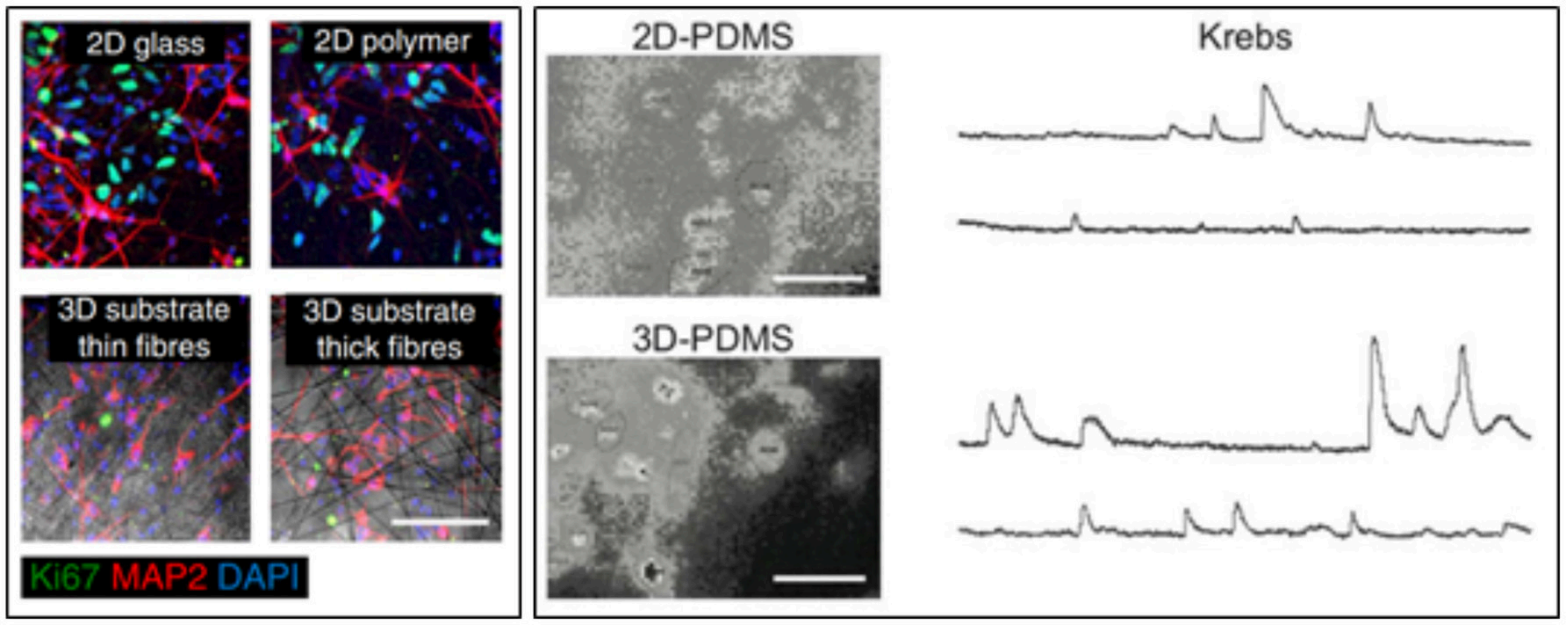
**(Left)** Human iN populations robustly express MAP2 in 2D and 3D conditions, while populations of unconverted, proliferative Ki67-expressing iPS cells persist in iN populations plated in 2D conditions. Scale bar, 100 mm (Carlson et al., 2016). **(Right)** snapshots of representative fields of neuronal cultures grown on 2D-PDMS (top) and 3D-PDMS (bottom) substrates, stained with the Oregon Green 488-BAPTA-1 AM. Scale bar: 50 μm. Repetitive Ca^2+−^events spontaneously recorded in hippocampal cultures of 9 DIV highlighted an higher frequency and synchronization of events in 3D cultures (Bosi et al., 2015).

The three-dimensional geometry crucially impacts neuronal network activity, while being reminiscent of the effective brain tissue architecture. It has been in fact demonstrated that neuronal ensembles were much more strongly synchronized and active if interfaced to a three-dimensional scaffold (Bosi et al., 2015; Carlson et al., 2016; Figure 8). This has been shown by using different types of scaffold materials, ranging from CNTs to the use of various types of polymer fibers (Bosi et al., 2015; Carlson et al., 2016). It was further demonstrated how grafting a scaffold containing Human induced Neuronal (iN) cells into the mouse striatum for 3 weeks led to a high percentage of viable cells one order-of-magnitude greater than that the grafting of isolated cells (Carlson et al., 2016).

## Conclusion

Nanoneuroscience integrates what is known about the nervous system and nanotechnology, two strongly progressing fields. The marriage of these two disciplines may provide a solution to many CNS disorders, from neurodevelopmental disorders to motor and sensory ones. In this review, we have reported about recent advances in nanotechnology for neural tissues. We described how neuroscience has increasingly applied nanotechnology strategies to develop innovative biocompatible nanotools, with the potential to enable more effective neural interfaces. Neuroscience and nanotechnology are thus poised to provide a rich toolkit of novel methods to explore brain function, by enabling the simultaneous measurement and the manipulation of activity of thousands or even millions of neurons.

## Author Contributions

NP, MG, DS, LB, and RR conceived, structured, and participated in writing the review.

### Conflict of Interest Statement

The authors declare that the research was conducted in the absence of any commercial or financial relationships that could be construed as a potential conflict of interest.
